# Center of Excellence in Research Reporting in Neurosurgery - Diagnostic Ontology

**DOI:** 10.1371/journal.pone.0036759

**Published:** 2012-05-14

**Authors:** Amrapali Zaveri, Jatin Shah, Shreyasee Pradhan, Clarissa Rodrigues, Jacson Barros, Beng Ti Ang, Ricardo Pietrobon

**Affiliations:** 1 Universität Leipzig, Institut für Informatik, Leipzig, Saxony, Germany; 2 Research on Research group, Duke University, Durham, North Carolina, United States of America; 3 Instituto de Cardiologia do RS, Porto Alegre, Brazil; 4 National Neuroscience Institute, Singapore; 5 Duke-NUS Graduate Medical School, Singapore; 6 Duke University, Durham, North Carolina, United States of America; University of Jaén, Spain

## Abstract

Motivation: Evidence-based medicine (EBM), in the field of neurosurgery, relies on diagnostic studies since Randomized Controlled Trials (RCTs) are uncommon. However, diagnostic study reporting is less standardized which increases the difficulty in reliably aggregating results. Although there have been several initiatives to standardize reporting, they have shown to be sub-optimal. Additionally, there is no central repository for storing and retrieving related articles. Results: In our approach we formulate a computational diagnostic ontology containing 91 elements, including classes and sub-classes, which are required to conduct Systematic Reviews - Meta Analysis (SR-MA) for diagnostic studies, which will assist in standardized reporting of diagnostic articles. SR-MA are studies that aggregate several studies to come to one conclusion for a particular research question. We also report high percentage of agreement among five observers as a result of the interobserver agreement test that we conducted among them to annotate 13 articles using the diagnostic ontology. Moreover, we extend our existing repository CERR-N to include diagnostic studies. Availability: The ontology is available for download as an.owl file at: http://bioportal.bioontology.org/ontologies/3013.

## Introduction

Evidence-based medicine (EBM) in neurosurgery relies on diagnostic studies and non-Randomized Controlled Trials (non-RCTs) since RCTs are uncommon. As a result there is ample controversy and lack of generalization which significantly impacts evidence-based practice. Aggregation of this evidence in the form of systematic reviews is a possible solution.

On the other hand, reporting is less standardized for diagnostic procedures, ultimately increasing the difficulty in reliably aggregating results [1]. This lack of standardization makes judgments about validity, bias, and applicability to patients in clinical settings difficult and in some cases impossible. When studies are poorly reported, contacting authors for missing information becomes necessary, thus increasing the probability of misreporting [2]. Although there have been several initiatives to creating reporting checklists and guidelines [3], [4], [5], reporting continues to be sub-optimal [1], [6].

In response to this problem, computational ontologies have been explored for RCTs [7], [8]. Ontologies contain controlled vocabularies which provide structured definitions and reasoning to terms from a particular domain. As one of its possible use cases, ontologies can enable standardized and semantically interconnected machine readable sections in a research article, ultimately enabling the semi-automated extraction of qualitative and quantitative information [9]. A previous effort towards an RCT ontology proposed the co-publication of RCT articles in prose and machine readable formats [7]. It was developed with a SR-MA (Systematic Reviews - Meta Analysis) use case in mind. Systematic reviews are studies that perform a literature review and then combine results of several studies in order to answer a particular research question. Meta-analysis, on the other hand, also combine results of several studies but based on statistical methods to identify an effect size. However, it was too comprehensive and included all terms specific for RCTs but also those terms not required for SR-MAs.

In comparison, Mesh (Medical Subject Headings) (http://www.ncbi.nlm.nih.gov/mesh/) and UMLS (Unified Medical Language System) (http://www.nlm.nih.gov/research/umls/) are controlled vocabulary thesaurus which are used for indexing and classifying articles. However, these were not developed with a specific use case whereas the diagnostic ontology is focused on stream-lining the process of conducting SR-MA for diagnostic studies. This paper is used to show that using ontologies, one is able to retrieve specific information, in an efficient and systematic way, needed to conduct the SR-MAs. It is also focused on diagnostic studies in neurosurgery as they are highly important in this field in order to formulate clinical practice guidelines. Ontologies not only help in integrating data from multiple sources in an interoperable way but also assist in querying and retrieving the specific required information easily.

Another issue that is of great concern to meta-analysts is searching for relevant literature. Despite their importance, the SR-MA process is slow, taking an average of two and half years to traverse from an RCT to a SR-MA. There are many instances when SR-MA have been known to take longer than nine years. In addition to the lack of enough publications to conduct a SR-MA, it is effort intensive and detail-oriented which makes it a tedious and time consuming task. To our knowledge, there is no central repository of diagnostic articles. Thus, we propose to extend our repository, namely CERR-N [10], to include articles of diagnostic studies to assist in streamlined and expedited SR-MA.

Although the inclusion of RCT ontologies has provided a significant step towards the improvement of reporting standardization, to our knowledge, previous studies have not reported the degree of observer agreement when attempting to annotate individual concepts in an article. This concept has been successfully tested with standardized checklists, demonstrating good reliability when assessed by observers of different experience and education levels [11], the agreement improving when a structured interview guide is used to train raters with little clinical experience [12].

Thus, in this article we will (1) describe the development of a diagnostic computational ontology to assist in diagnostic article standardization, (2) share the results of an interobserver agreement test to validate the ontology’s accuracy and consistency in tagging a manuscript and (3) describe the extension of CERR-N to include diagnostic studies.

## Results

### Ontology Structure

**Figure 1:**
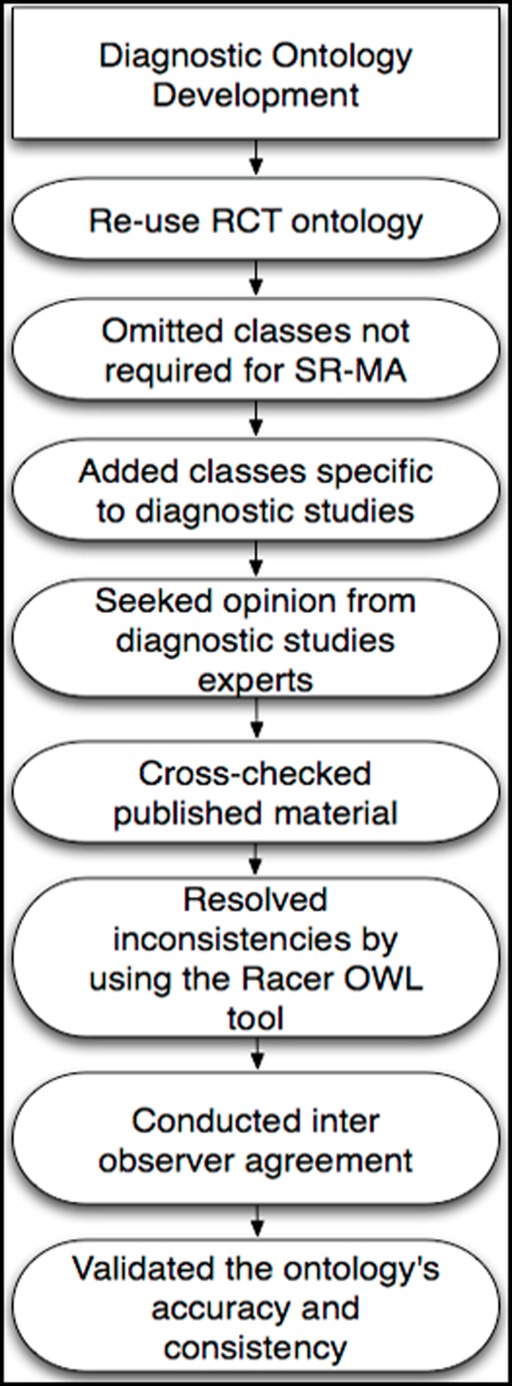
Steps involved in designing the diagnostic ontology.

The diagnostic ontology consists of 91 elements, including classes and sub-classes, which are required to conduct SR-MA for diagnostic studies. The hierarchy is displayed in Figure 1. Compared to the RCT ontology, there are 37 elements less as those concepts (or classes) are not considered while conducting diagnostic studies. There were a number of new classes that were added such as the sub-classes under AssesmentRiskBias to cover all the risks and biases that occur in such studies. The ontology contains a hierarchy representing class and sub-class relationships between the classes, which means that any instance of a sub-class is automatically an instance of the parent class. The classes were also specified to be disjoint and certain restrictions were specified for classes that take only certain values as input for their instances. The validation of the ontology was done by using the SPARQL query language, that would allows us to retrieve instances of the classes based on the information we require.

### Extension of CERR-N

CERR-N is a repository of standardized articles, all related to neurosurgery. The standardization is aimed to streamline the SR-MA process. In our previous paper [10], we described the inclusion of RCTs in this repository, which are standardized using the RCT ontology. With the diagnostic ontology in place, we will extend the repository to also include diagnostic articles. With this addition, the repository will facilitate researchers’ reliance on a central place within which to search for articles of interest to them. All articles present in the repository will be tagged using a particular keyword, as described in our previous paper. Thus, extracting articles will be much faster and thus meta-analysts can then proceed to conduct SR-MA after retrieving the relevant articles. Updating an existing SR-MA will also be possible, thereby providing additional functionality to the repository.

### Use Case

As a use case we describe the steps involved in performing a systematic review and meta-analysis of diagnostic studies. The first step involves retrieval of the fields required for a qualitative comparison across the papers. This step is essential for assessing the heterogeneity across the articles and to decide which ones to include in the quantitative analysis. The next step is to retrieve fields for the quantitative comparison across the papers selected from the first step. Then, the appropriate statistical values are fed into a meta-analysis software to perform the meta-analysis. As an example, we will describe the steps involved in performing a meta-analysis of diagnostic studies for the topic “back pain.” First, the researcher queries the repository to retrieve all articles with the tag “back pain” and retrieves all fields required for a qualitative comparison across the papers, as recommended by the Cochrane Collaboration (http://cochrane-handbook.org/(Section 11.2)).

The data is queried using the SPARQL (SPARQL Protocol and RDF Query Language) query language which is able to retrieve and manipulate data stored using an ontology. It is a W3C standard (http://www.w3.org/TR/rdf-sparql-query/), which has a syntax almost similar to the SQL query language, except the variables are indicated by a “?”. The SELECT query is utilised to extract raw values from a dataset and the results are returned in a table format. The envisioned SPARQL query for our use case is illustrated in Listing 1.

prefix: doc:<http://neurosurgery.org/document/>

SELECT *

WHERE {

?document doc:title “back pain”.

?document doc:has_name ?trial.

?document doc:method ?MaterialMethods.

?document doc:type ?TrialDesign.

?document doc:population ?AnalyzedPopulation.

?document doc:intervention ?Procedure.

?document doc:result ?ConclusionDetails.

Listing 1. SPARQL query retrieving summary data required for quantiative comparison

prefix: doc:<http://neurosurgery.org/document/>

SELECT *

WHERE {

?document doc:has_name ?trial.

?document doc:population ?AnalyzedPopulation.

}

Listing 2. SPARQL query retrieving summary data required for quantiative analysis

This query generates a table with values, which can be used as input in a meta-analysis software, such as RevMan, to calculate the treatment effect or effect size of all the studies. The researcher can then decide the appropriate meta-analysis method to be used, based on the recommendation by Cochrane (Table 9.4.a in the Cochrane Handbook) according to the type of data (e.g. dichotomous, continuous).

### Interobserver Agreement

The overall percent agreement among the raters was high (Table 1). This is associated with a fair to poor level of agreement according to the Landis and Koch scale [13]. Amongst the 13 articles the percent agreement was high (82.85%) for article 9.

**Table 1:** Interobserver agreement.

Article	Percent agreement (%)
1	47.22
2	53.04
3	48.57
4	48.75
5	56.87
6	60.00
7	54.54
8	62.94
9	82.85
10	71.11
11	70.90
12	78.18
13	66.00

## Discussion

We developed and validated a biomedical ontology, focused on diagnostic studies, with a well defined use-case aimed at streamlining meta-analysis of such studies. Since end users form an integral part of our proposed use-case, we believe that accurate annotation of diagnostic studies is essential for the successful use of the ontology. Accordingly, we evaluated interobserver agreement for the ontology class annotations implemented in a sample of 13 diagnostic studies and noted a reasonable degree of agreement amongst observers.

Although SR-MAs form an important part of evidence-based practice and policy, the number of SR-MA publications is lower than their corresponding demand. This demand-supply gap exists not only on account of the time and effort intensive nature of SR-MAs, but also due to the lack of standard and transparent reporting guidelines for primary studies. The former requires significant time commitment from SR-MA researchers while the latter makes it difficult to combine results from primary research studies. Although the introduction of reporting guidelines and standards have partially resolved the issue, the quality of scientific reporting continues to be below expectations [14], [15], [16]. In the context of diagnostic studies, the prevailing lack of transparency in reporting was addressed by the introduction of the STARD guidelines in 2003. Recent reports suggest that widespread conformance is yet to be achieved among journal articles and quality of reporting is similar between the pre STARD and post STARD implementation phase [6]. With the increase in number and type of SR-MAs there is a need to refine the indexing, which can in turn permit efficient search, retrieval and organization of SR-MAs [17]. Although MESH terms (http://www.nlm.nih.gov/mesh/), Limits (http://www.ncbi.nlm.nih.gov/pubmed/limits) and SR-MA filters are some solutions introduced in PubMed (http://www.ncbi.nlm.nih.gov/pubmed/) and other electronic databases, SR-MA conduct continues to remain a time consuming exercise. Lastly, even though it is essential to facilitate the maintenance and update of SR-MAs especially after the publication of new evidence and criticism [17], outdated SR-MAs continue to mislead readers and end up becoming the basis of policy guidelines. Synthesizing new evidence, corrections, criticisms and responses is a tedious task in itself especially when these are widely scattered [17].

Biomedical ontologies are seen as a possible solution to these issues as well as to semi-automate the SR-MA process. The fundamental role of ontologies is to share and re-use the knowledge which it represents. Ontologies have the ability to classify, categorize and define a hierarchy of concepts and the relationships existing between them. As a result they can facilitate structured reporting and help in extracting information needed to conduct a SR-MA. They also allow efficient search, indexing and reasoning of data and thus in principle can help in the expediting the conduct and update of SR-MAs. The Trial Bank Ontology [7] was developed with SR-MA use case in mind. Another study developed by our group [9] showed that this ontology was able to closely represent the findings from the original meta-analysis with decreased time requirements of the manuscript author and the software programmers. However, this ontology included many terms not required for SR-MA, and also this ontology only focused on RCTs, while we focus on diagnostic studies since these are commonly used in neurosurgery. The use of this ontology could improve the speed with which a SR-MA is performed and could potentially help in standardizing the elements required within a diagnostic study.

Although computational ontologies are often referred to as a mechanism to reduce the number of conflicting definitions among different users [18], a formal testing of whether an improvement in agreement occurs is rarely performed. This gap is surprisingly present even in areas where the issues associated with lack of observer agreement have been well documented, such as in the case of ontology-assisted meta-analyses. We found significant agreement among the observers who annotated the diagnostic articles. Although these results are not generalizable, they indicate a possibility of easier adoption and use among naive researchers if utilized and implemented by scientific research journals.

High quality ontologies are a very important contribution to the standardization of reporting of studies. However, there are a number of problems that continue to effect their reliability. Studies evaluating ontologies reveal several areas from which errors tend to emerge including philosophical rigor, ontological commitment, content correctness and fit for purpose [19], which can impact the interoperability of ontologies. While these problem areas are increasingly addressed through quality assurance systems, a surprising issue that has been largely overlooked is whether ontologies actually fulfill their intended purpose of reducing the number of conflicting definitions among different users [18].

We intend to further develop this line of research following on the present study in the following ways: (1) We plan to evaluate our ontology by applying it to several diagnostic studies and measuring the effectiveness and efficiency of using ontologies for performing meta-anlalysis; (2) We plan to create an ontology for non-RCTs since these studies are also considered to be important for EBM; (3) We aim to link the ontologies to peer-reviewed journals so as to make it mandatory for researchers to use the ontology structure to format the articles while submitting; (4) We plan to extend CERR-N to include articles not only in neurosurgery but also in other areas of medicine.

In conclusion, we have described the development of a diagnostic ontology to standardize reporting of diagnostic studies as well as streamline the meta-analysis process. We have also included details of the extension of the CERR-N repository which will store all articles tagged using the ontology and thus facilitate in retrieving similar articles easily and faster to conduct meta-analysis. A use case is also presented illustrating the process involved in performing the meta-analysis of diagnostic studies.

## Methods

**Figure 2:**
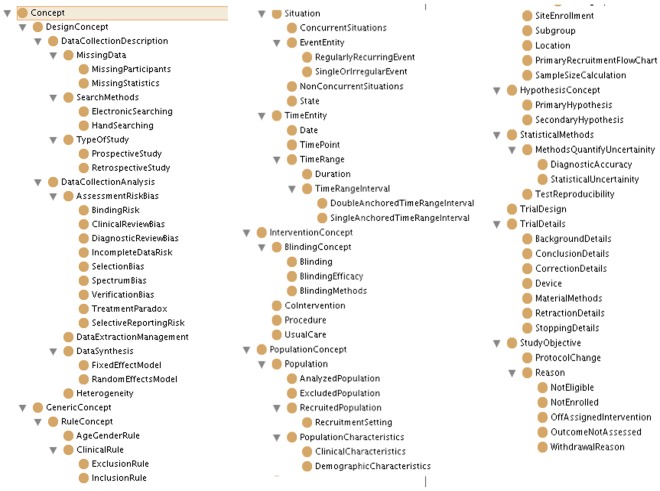
Hierarchy of classes present in the diagnostic ontology.

In our approach, we used the standard ontology engineering steps to design the ontology [20], [7], [8]. In particular, we first re-used the RCT ontology by analyzing and omitting classes not required for SR-MA of diagnostic studies. Subsequently, we added classes that were essential for diagnostic studies. We not only sought the opinion of experts in diagnostic studies regarding the classes of the ontology but also cross checked published material to ensure the completeness of the ontology. Thereafter, we resolved inconsistencies that were encountered during tests. As an effort to validate the usefulness of the ontology, we calculated the interobserver agreement between five observers who tagged 13 articles with the ontology. Figure 2 illustrates the steps that we followed while designing the diagnostic ontology.

### Ontology Development

In this section we describe in detail the steps that we followed to develop the diagnostic ontology, by following standard ontology engineering steps.

#### Re-use RCT Ontology

We used the RCT ontology previously developed by our group [10] as a template to work towards developing the ontology for diagnostic studies. This was done to follow the principle of re-using an existing ontology while creating a new one [20]. The RCT ontology consists of 128 elements, but since not all of them are required for diagnostic studies, we analyzed each class and its sub-classes to determine whether to keep or omit that concept.

#### Omitted classes not required for SR-MA

In this step, we omitted those classes (concepts) not present in diagnostic studies which are listed in Table 2.

**Table 2:** Classes omitted from the RCT ontology.

Class/Concept Omitted	Reason
Secondary Study	Additional investigation pertaining the same interventions as the primary study.
FraudDetails	The organization or institution that verified the fraud, if present. Since this class was not amongst the classes used in RCT ontology.
Situation	Since this class was not amongst the classes used in RCT ontology.
PopulationConcept	Extensively modified to exclude certain sub-classes.
ProtocolConcept	Description of the objective(s), design, methodology, statistical analysis, and organization of a trial.
OutcomeConcept	Extensively modified.
InterventionConcept	Extensively modified.

#### Added classes specific to diagnostic studies

After omitting classes that were not required for diagnostic studies, we also added certain concepts to the ontology that were essential for conducting SR-MA of diagnostic studies. These classes or concepts are represented in Table 3.

**Table 3:** Classes added in the diagnostic ontology.

Class/Concept Added	Reason
AssessmentRiskBias	Any kind of bias introduced in the study affects the results, thus it is important to take this concept into account while aggregating several studies.
DataExtractionManagment	The method used to extract or obtain data from published reports or from the original researchers (for example, using a data collection form) is noted in this concept. Whether the data is extracted independently by more than one authors is also noted, along with how any disagreements are resolved. If relevant, the description of the methods for processing data in preparation for analysis is also mentioned.
Heterogeneity	Since variability is the rule rather than the exception, researchers should explore possible sources of heterogeneity in results, within the limits of the available sample size. Thus this concept is introduced in the ontology.
DataCollectionDescription	This concept is introduced to assess whether the data collection was planned before the index test and whether the reference standards were performed (such as in a prospective study) or after (such as in a retrospective study).
StatisticalMethods	In order to obtain information about the statistical methods used during the study, this concept is introduced.

#### Opinion seeked from diagnostic studies expert

In order to be representative of the latest information in the field as well as to enhance its utilization among institutions currently conducting SR-MAs of diagnostic studies, we engaged an expert in diagnostic studies (IC) as well as an active member of the department of Public Health and Medical Informatics at Université Paris V and Hôpital Européen Georges Pompidou. This ensured that all elements essential for a meta-analysis were included in the ontology.

**Table 4:** Articles Included.

Journal	Article Title	Year of Publication
Journal of Neurology, Neurosurgery and Psychiatry	Real time polymerase chain reaction: a new powerful tool for the diagnosis of neurobrucellosis (Colmenero et. al.)	2005
Journal of Neurology, Neurosurgery and Psychiatry	Diagnostic value of the Rotterdam-CAMCOG in post-stroke dementia (Koning et. al.)	2005
Journal of Neurology, Neurosurgery and Psychiatry	Comparison of intra-arterial thrombolysis with conventional treatment in patients withacute central retinal artery occlusion (Arnold et. al.)	2005
Journal of Neurology, Neurosurgery and Psychiatry	Sensitivity and specificity of the new international diagnostic criteria for migraine withaura (Eriksen et. al.)	2005
Neurosurgery	Limitations of magnetic resonance imaging and magnetic resonance Angiography inthe diagnosis of intracranial aneurysms (Schwab et, al.)	2008
Neurosurgery	Comparison of two techniques to postoperatively localize the electrode contacts usedfor subthalamic nucleus stimulation (Pinto et. al.)	2007
Neurosurgery	Positron emission tomography with O-(2-[18F]fluoroethyl)-L-tyrosine versus magnetic resonance imaging in the diagnosis of recurrent gliomas (Rachinger et. al.)	2005
Neurosurgery	Detection of intracranial aneurysms with two-dimensional and three-dimensionalmultislice helical computed tomography angiography (Kangasniemi et. al.)	2004
Journal of Neurosurgery	Sixteen-row multislice computed tomography angiography in the diagnosis and characterization of intracranial aneurysms: comparison with conventional angiographyand intraoperative findings (Chen et. al.)	2008
Journal of Neurosurgery	Endoscopic management of arachnoid cysts: an advancing technique (Karabatsou et. al.)	2007
Journal of Neurosurgery	Frameless image-guided stereotactic brain biopsy procedury: diagnostic yield, surgical morbidity, and comparison with the frame-based technique (Woodworth et. al.)	2006
Journal of Neurosurgery: Spine	A new concept in the electrophysiological evaluation of syringomyelia (Roser et. al.)	2008
Annals of Neurology	Assessment of nerve degeneration by gadofluorine M-enhanced magnetic resonanceimaging (Bendszus et. al.)	2005

#### Cross checked published material

We referred to the Cochrane Handbook for Systematic Reviews of Intervention [21]; reviewed to published material [22], [2]; went through the check-list [23] focused on diagnostic studies and analyzed papers of this design from neuroinformatics journals to cross-check whether any elements were missed. We compared our initial draft of classes and relationships against the elements present in STARD guidelines. Specifically, we aligned the classes so that previous articles created based on the STARD guidelines, can be aligned with our proposed diagnostic ontology.

#### Resolved inconsistencies

In order to evaluate the ontology, we used the Racer OWL tool (Racer Systems GmbH and Co. KG, 2004) in Protege to resolve the inconsistencies identified by the tool. This was essential to check if the constraints and relationships specified in the ontology gave appropriate results when queried. A consistency check was also done to ensure that the ontology does not include any contradictions. We ensured the the ontology is internally consistent by populating the ontology with instances from a diagnostic article (Table 4). We checked for inconsistencies in the classes and class hierarchy; class properties such as objective and datatype properties, domain, range, disjointness amongst sub-classes; cardinality, values, relationships and restrictions.

#### Calculated Interobserver Agreement

The Journal of Neurology, Neurosurgery and Psychiatry, Annals of Neurology and The Lancet Neurology journals were hand-searched to retrieve the diagnostic studies published within the last five years (2004–2008). The following operational definition served as guidelines to include or omit studies in our sample. *Diagnostic studies are those studies that test a new diagnostic method and compare it with a ‘gold standard’ method of diagnosing a disease*. Such studies should include details about (a) index test (b) reference test and its rationale; (c) study population including inclusion and exclusion criteria; (d) participant recruitment and sampling; (e) data collection; blinding method used; (f) defined primary and secondary outcome measures; (g) statistical methods and results; (h) estimates of diagnostic accuracy and measures of statistical uncertainty [23]. A sample of 13 articles (Table 4) were selected from the three journals. Five observers (AP, AZ, MV, SuP, SP) were chosen for the interobserver agreement test, of which two had a background in ontologies (AZ and SP) while the others had a clinical research background (AP, MV, SuP). They tagged all 13 articles using the ontology in Word documents by writing the tag name in square brackets against the word/phrase/sentence in the article. We then calculated the interobserver agreement amongst the five observers for the 13 articles and have reported them as percentages (Table 1).
